# Don't Miss the Well-Being Train: A Radical Proposal for Revolution in Positive Psychology

**DOI:** 10.3389/fpsyg.2021.794065

**Published:** 2021-12-23

**Authors:** Dan Weijers

**Affiliations:** School of Social Sciences, University of Waikato, Hamilton, New Zealand

**Keywords:** positive psychology, well-being, Kuhn, paradigm-shift, reverse shark tank, model consolidation, interdisciplinary

## Introduction

Positive psychologists have added a great deal of knowledge about positive mental states and behaviors, especially over the last 20 years. As evidence is produced in support of the connections between positive psychological constructs, the model of mental flourishing is slowly being filled in. But, just like the broader well-being research and policy community (Lee et al., 2021), the discipline remains divided about how to conceptualize and measure well-being (Hone et al., 2014; Ackerman et al., 2018). This lack of unity makes it difficult to establish how measures of positive mental functioning connect with the bigger picture of the good life for the one living it, and deter its use in broader research and policy agendas. For example, the influential World Happiness Report 2020 (Helliwell et al., 2020) is edited by only economists and contains the phrase Positive Psychology just once.

As more and more governments prioritize well-being, knowing how to measure well-being has never been more important. Unless positive psychologists can quickly converge on a view about what well-being is, how positive psychological constructs connect to well-being, and the best practices for measuring these, they may not have the influence they deserve on the policymaking agenda. Instead, economists, demographers, statisticians, and the status quo will have the most influence on which measures become entrenched as the ways to conceptualize and measure well-being. This has certainly been the case in Aotearoa New Zealand. The input into and commentary on the Living Standards Framework (the fundamental well-being policy framework for the nation) has overwhelmingly been from economists, then statisticians, with very minor roles played by demographers, philosophers, and positive psychologists.

For Positive Psychology to help model well-being and steer public policies in a positive direction, urgent interdisciplinary work is needed. Modeling and measuring well-being require the proper conceptualization of the concept in the first instance. I propose that positive psychologists work with philosophers and other social scientists to achieve this conceptual clarity. The next step is to establish the criteria for a good measure of well-being, with extra criteria for various use-cases, especially policymaking. Again, this should be an interdisciplinary endeavor. Finally, interdisciplinary teams should evaluate proposed measures of well-being.

A major concern for Positive Psychology in achieving all of this is the potential reluctance of established figures to allow progress away from the extant psychological scales and models of well-being. To combat this, I propose a highly unorthodox method that would take advantage of the wealth of knowledge established figures have, while passing the gatekeeper role to the next generation of researchers—a reverse shark tank.

## Revolutionary Change

As Kuhn (1962) noted, scientific progress is usually incremental and bound within its dominant paradigm. For Positive Psychology, that might mean validating Diener's Satisfaction with Life Scale (Diener et al., 1985) in a new culture or devising a set of items that more accurately measures positive affect. More productive periods of scientific progress, according to Kuhn (1962), tend to follow from paradigm shifts—big changes in underlying models or dominant methods. Positive Psychology's current paradigm appears to be focussed on conceptualization, generation, and validation, resulting in the proliferation of models and partial models of human well-being (see e.g., Lee et al., 2021). A unification and rationalization period (similar to what happened in personality research and resulted in the “Big Five”; John and Srivastava, 1999) is the obvious next step. We need a paradigm shift in Positive Psychology to consolidate the decades of successful research into a unified model or set of models (for various purposes). Importantly, methodological issues, including dealing with cultural differences and ordinal scales also need to be addressed in this process, with clear and agreed upon guidelines for minimum viable and best practice.

Such a paradigm shift will not be easy to achieve, but it could be hugely important. If Positive Psychology can unify around a model of well-being and work out which scales best integrate with it, cross-fertilization of research programmes should be easier. Furthermore, other disciplines and policymakers are more likely to consider elements of Positive Psychology settled enough to inform their work. Assuming the work to unify around a particular model of well-being is deeply informed by interdisciplinary researchers and policymakers, those other groups are also much more likely to use the insights of Positive Psychology to guide their work.

## Gate-Keeping in Positive Psychology and Beyond

As I mentioned, this paradigm shift will not be easy. An under-discussed but potentially important obstacle to Positive Psychology achieving this radical change is the possible reluctance of established figures to allow progress away from the scales and models they have created.

On the pragmatic side, the established scales have already been extensively validated and the dominant models extensively used. We already know how the scales operate in various cultures and what other variables of interest they tend to predict. We also have benchmarks of the dominant models to compare new results against. I know from discussions with tight-budgeted national statisticians that the pragmatic draw of established measures and models is very strong. But this pragmatic issue might not be the only barrier for a new unified model of well-being to overcome.

Kuhn (1962) identified established academics as a major obstacle to scientific paradigm shifts. The senior professoriate of Positive Psychology are scientific gatekeepers in many ways and the decisions they make can be affected by subconscious biases. Senior positive psychologists hold great editorial power over publication decisions, but can be biased against unfamiliar or novel models and methods due to confirmation bias (Greenwald et al., 1986; Solomon, 2001; Jelicic and Merckelbach, 2002), perhaps driven by availability and overconfidence bias (Dunning, 2005; Rollwage et al., 2020). And since confirmation bias is directed at ideas, rather than people, even triple-blind review processes cannot protect from it. Consider a senior editor at a Positive Psychology journal receiving a manuscript that uses a methodology they are unfamiliar with; feeling uncertain about the quality of the methodology, they may send it to a reviewer that they know has very high standards. While well-meaning, decisions like these may raise the bar for novel and potentially revolutionary research to be published. The editor and reviewer may well share similar views about which topics and methods are important. As discussed by Lee et al. (2013), when the reviewer submits their own paper to the editor's journal, the editor might send the paper to reviewers with lower standards as a way to thank he reviewer for their service, thereby potentially lowering the bar for authors that are more likely to shared views with the editor. Senior positive psychologists also attract and direct most of the grant funding. While, I assume, the vast majority of people in these roles try to be impartial, Lacey (1999, p. 6) has argued that they cannot help but be swayed by the topics they are interested in, the methods they are familiar with, and perhaps even “expedient alliances” with other senior researchers. These biases provide several reasons for why powerful academics might make decisions that help ensure that their scales and their models continue to be the focus of any attempt to unify Positive Psychology. It is difficult to gauge, with any precision, the extent to which these biases are affecting the progress in Positive Psychology specifically. Nevertheless, the wealth of evidence supporting the existence of biases that can affect the progression of science, gives us reason to take Kuhn's (1962) warnings seriously. But how should Positive Psychology deal with this barrier to fruitful unification?

Kuhn (1962), again, is instructive. The greatest enemy to senior academics is time. Fatigue, ill-health, and occasionally other interests or demands lead senior academics to retire and relinquish their roles as gate-keepers. We (with regret^1^) currently find ourselves in a period of incremental leadership change. The original pioneers of Positive Psychology are, one way or another, passing the gate-keeper roles onto successors. The successors, of course, are also mainly hand-picked very well-known positive psychologists that studied or worked with the outgoing leaders. None of this is sinister. Rather, it is sociologically and psychologically natural, and certainly expected. The main issue for Positive Psychology is that its relevance to the overall well-being research and policy discussions needs to be demonstrated soon if Positive Psychology is to be meaningfully involved. It would be a huge risk to wait for incremental leadership change that may only result in superficial incremental change, rather than fundamental scale and model consolidation. So, what should be done?

Naturally, major funding is required. Previous major funding has been directed at the goal of scale and model unification. A few major projects have fruitfully brought together Positive Psychologists and others to—among other goals—debate how to conceptualize and measure well-being. A good example of a general project is Haybron's Happiness and Well-Being project (https://www.happinessandwellbeing.org/). A good attempt at reaching interdisciplinary consensus on conceptualizing and measuring well-being is reported on in Lee et al. (2021). Lee et al.'s contribution includes a sustained debate between groups of experts about the merits of different approaches to measuring well-being. No consensus was reached in the end, but the process and the arguments involved are instructive for all well-being researchers. Presumably the gate-keeper effect of the senior well-being researchers involved had some influence on how the discussion evolved, and possibly decreased the chances that consensus could be reached. So, it seems that major funding is not enough by itself. But what else is required? In particular, if the entrenched views and power of senior Positive Psychologists is a barrier to paradigm-shifting scale and model consolidation, how can we overcome this?

## Reverse Shark Tank

The wisdom of Positive Psychology's current leaders can be harnessed in a way that promotes paradigm-shifting scale and model consolidation, but it will not be easy and it may require a highly unorthodox approach.

In the popular television show, Shark Tank, contestants pitch their business ideas to established angel investors—gatekeepers of the business world—with successful pitches resulting in funding offers. The way most funding opportunities work in Positive Psychology is similar—senior positive psychologists act as gate-keepers, often advising on or deciding which grant applications are funded. Some problems with this approach, including how it favors established approaches and incremental scientific progress, were discussed above. But what if the process were reversed?

Imagine a process that had funders and an interdisciplinary team of early/mid-career researchers as the reviewers and deciders. In this “reverse shark tank,” interdisciplinary teams, including many led by senior researchers, would pitch their ideas for unifying scales and models of well-being to gatekeepers that have fewer reasons to resist revolutionary paradigm-shifting change in Positive Psychology. The relatively young gatekeepers in the reverse shark tank would be much less likely to be deeply attached to existing scales and models because they are less likely to be the authors of them or have staked their careers on them. For the same reason, the new gatekeepers would be more likely to support the ongoing research programme based on the process. They would also have much of their research careers left to do so. In this way, the reverse shark tank process harnesses the great wealth of expertise held by the current leaders of Positive Psychology, while reducing the psychological and sociological impediments to revolutionary change that appear to have been holding Positive Psychology back from having a greater influence in wider well-being related research and policy.

Ideally, the pitting of leaders against other leaders would encourage meaningful cooperation and compromise between leaders with different approaches and leaders from different disciplines as they strive to make the most compelling pitches. Clearly stating the goal of the reverse shark tank process as revolutionary change to create best practice scales and a unified model of well-being should also deter incremental business-as-usual proposals and promote genuinely novel approaches.

## Conclusion

Nothing I've said here is meant blame senior academics for what are merely common sociological and psychological forces. Nor have I meant to belittle or ignore the various projects that have attempted and in various ways succeeded in making progress in scale and model development, or in influencing public policy (I certainly cannot mention them all here, but a good recent example would include the various projects running out the Centre for Positive Psychology in Melbourne, such as the well-being literacy project; Oades, 2017). Rather, I hope this will be seen as a call for Positive Psychology and its friends to redouble efforts to make our research more robust and useful for practical purposes. This hope is based on my views that Positive Psychology has so much to offer everyone and that it would be a shame if that opportunity was not pursued to its fullest extent. And while I know my Kuhn-inspired sociological reflections and reverse shark tank idea were brief and possibly idealistic, I hope they can encourage Positive Psychology to flourish in this way.

## Author Contributions

All research and writing performed by DW.

## Funding

A FASS Research Grant (#107526) from the University of Waikato covered the article processing fee.

## Conflict of Interest

The author declares that the research was conducted in the absence of any commercial or financial relationships that could be construed as a potential conflict of interest.

## Publisher's Note

All claims expressed in this article are solely those of the authors and do not necessarily represent those of their affiliated organizations, or those of the publisher, the editors and the reviewers. Any product that may be evaluated in this article, or claim that may be made by its manufacturer, is not guaranteed or endorsed by the publisher.

## Footnotes

^1^Please understand that I am not trying to say anything negative about any particular individuals. In particular, I mean no disrespect to recently retired or especially recently deceased colleagues.
